# Preliminary Validation of the Greek Version of the Cervical Cancer Prevention Knowledge (CCKP) Questionnaire

**DOI:** 10.7759/cureus.113985

**Published:** 2026-08-05

**Authors:** Aikaterini Toska, Dimitra Georga, Dimitra Latsou, Evangelos C Fradelos, Erasmia Rouka, Theodosios Paralikas, Pavlos Sarafis, Konstantinos Tsaras, Dimos Sioutis, Maria Saridi

**Affiliations:** 1 Department of Nursing, University of Thessaly, Larissa, GRC; 2 Department of Business Administration, University of West Attica, Athens, GRC; 3 Department of Nursing, University of Thessaly, Lamia, GRC; 4 Third Department of Obstetrics and Gynecology, Medical School, National and Kapodistrian University of Athens, Attiko Hospital, Athens, GRC

**Keywords:** cervical cancer, cultural adaptation, greece, knowledge, questionnaire validation, reliability

## Abstract

Background and aim

Women’s perceptions and knowledge of cervical cancer have a significant impact on the adoption of preventive measures. Nonetheless, there is limited information on the availability of culturally adapted and psychometrically validated tools to measure cervical cancer prevention knowledge in different populations, including Greece. This study aimed to translate, culturally adapt, and evaluate the face validity, content validity, and test-retest reliability of the Greek version of the Cervical Cancer Prevention Knowledge (CCKP) questionnaire among women in Greece.

Methods

This methodological study involved the adaptation of an existing measurement tool, following standard procedures described in the international literature on translation and cultural adaptation. Face validity was evaluated qualitatively by five experts and quantitatively in 15 women from the target population using the item impact score. Content validity was assessed by the expert panel (five experts) using the Item-Level Content Validity Index (I-CVI), Scale-Level CVI (S-CVI)/Ave, S-CVI/UA, and modified kappa statistics. Reliability was examined using the test-retest method and internal consistency analysis in a sample of 50 women. Agreement was assessed by calculating unweighted Cohen’s kappa, while internal consistency was evaluated using Cronbach’s alpha and the Kuder-Richardson Formula 20 coefficient.

Results

The Greek version of the CCKP questionnaire demonstrated satisfactory face and content validity, with high I-CVI and S-CVI values indicating strong expert agreement regarding item relevance. All items met the predefined quantitative face validity threshold. I-CVI values were 0.80 or 1.00, S-CVI/Ave was 0.97, and S-CVI/UA was 0.84. Modified kappa values were 0.76 or 1.00. Percentage agreement ranged from 62.0% to 98.0%, while unweighted Cohen’s kappa values ranged from 0.56 to 0.97, indicating moderate to almost perfect agreement. Some adjustments to the questionnaire enhanced its cultural and content validity. Internal consistency coefficients for the evaluated domains ranged from 0.730 to 0.884.

Conclusions

The Greek version of the CCKP questionnaire demonstrated acceptable face validity, content validity, and test-retest reliability for assessing cervical cancer prevention knowledge among women in Greece. Further evaluation of construct validity, criterion validity, and factor structure is required in larger and more diverse samples.

## Introduction

Cervical cancer poses a significant threat to global public health, although it is one of the most preventable cancers through primary and secondary prevention. In 2022, approximately 660,000 women were diagnosed with cervical cancer worldwide, and about 350,000 died from the disease [1]. Infection with human papillomavirus (HPV) is recognized as the leading cause of cervical cancer, whereas early detection through the Papanicolaou test and HPV vaccination programs helps reduce its incidence and mortality [2,3].

Although effective preventive interventions exist, participation rates in cervical cancer screening programs remain low. Knowledge, attitudes, and beliefs play critical roles in women’s decisions to adopt preventive health practices [4,5]. Thus, accurately assessing these factors is essential for developing appropriate public health interventions. Various research instruments are available for this purpose, among which the Cervical Cancer Prevention Knowledge (CCKP) questionnaire has gained wide acceptance. This instrument comprises several thematic domains covering general knowledge, risk factors, primary and secondary prevention, and sources of information about cervical cancer.

Nevertheless, implementing research instruments in different cultural and linguistic settings requires a systematic translation and cultural adaptation process to ensure that the instrument remains valid, in accordance with the recommendations of Beaton et al. (2000) and Sperber (2004) [6,7]. The use of untranslated or inadequately validated instruments may compromise the accuracy of findings and limit comparisons across studies.

Research examining Greek women’s knowledge and perceptions of cervical cancer remains limited, and no culturally adapted and psychometrically evaluated Greek version of the CCKP questionnaire has been identified. Against this background, this study aimed to translate, culturally adapt, and evaluate the face validity, content validity, and test-retest reliability of the Greek version of the CCKP questionnaire among women in Greece.

## Materials and methods

Study design

A methodological study was conducted to translate, culturally adapt, and preliminarily validate the Greek version of the CCKP questionnaire. Specifically, the study assessed face validity, content validity, test-retest reliability, and internal consistency. The study was conducted in three main stages: (1) translation and cross-cultural adaptation, including pilot testing; (2) assessment of face and content validity; and (3) evaluation of test-retest reliability and internal consistency.

Experts participating in the content validity assessment were purposively recruited from academic and clinical departments affiliated with the University of Thessaly based on their professional expertise and experience in cervical cancer, public health, nursing, and questionnaire validation. Women participating in the test-retest reliability assessment were recruited using a nonprobability snowball sampling approach. Eligible participants were women aged 21-69 years who were able to understand written Greek and independently complete the questionnaire. This age range was selected to include women at different stages of adulthood and with potentially different experiences of cervical cancer prevention and screening. Initial recruitment was conducted through social media, printed study materials, and the researchers’ professional and personal networks. Participants were subsequently invited to share the study invitation with other eligible women. This approach facilitated the recruitment of women from different geographical areas and socioeconomic backgrounds across Greece, although it may have limited the representativeness of the sample and increased the risk of selection bias. Data were collected nationwide between June and November 2025.

Ethical considerations

The study was conducted in accordance with the principles of the Declaration of Helsinki. Ethical approval for this study was obtained from the Ethics and Deontology Committee of the Department of Nursing, University of Thessaly (approval 391). Participants were informed about the purpose of the study and provided written informed consent before participation. Participation in the study was voluntary and anonymous.

Description of the original questionnaire

The questionnaire was developed by Jaglarz et al. (2014) to assess knowledge of cervical cancer prevention [8]. It consisted of closed-ended questions and was divided into five domains according to guidelines adapted from the European Organisation for Research and Treatment of Cancer (EORTC). Permission to translate, culturally adapt, and use the CCKP questionnaire was obtained from the original developers before the commencement of the study.

The first domain assesses general knowledge of cervical cancer prevention through six questions. The second domain relates to the association between cervical cancer and specific risk factors. In this section, 17 factors were listed, and participants were asked to rate each factor on a six-point Likert scale, where 0 represented no perceived association and 5 represented a very strong association with cervical cancer. The third domain measured knowledge of primary prevention of cervical cancer and was divided into three subdomains: (A) lifestyle, (B) vaccination, and (C) optimal age for HPV vaccination. The fourth domain assessed knowledge of secondary prevention and consisted of two subdomains. Subdomain A investigated the perceived relationship between the listed symptoms and cervical cancer, and subdomain B measured knowledge regarding the Pap test. The fifth domain explored the sources of information used by respondents to learn about cervical cancer. The CCKP questionnaire has been applied in previous studies [2-5,9].

Translation

The source questionnaire was developed in English and translated into Greek. The translation and cross-cultural adaptation process followed a bidirectional approach, consisting of forward and backward translation, review by an expert committee, and preparation of the final version for pilot testing. Cross-cultural equivalence was ensured by focusing on language comparability, interpretive consistency, and conceptual understanding across the different versions of the instrument. The translation process included all questionnaire items and the accompanying completion instructions and was guided by a six-step translation framework that allows for empirical validation [6].

The forward translation was performed independently by two native Greek speakers, both faculty members in a nursing department with substantial familiarity with the cervical cancer literature. The translators independently translated the original questionnaire into Greek and reviewed the item wording, making modifications when necessary to enhance the cultural relevance of the items within the Greek context. The resulting version was written in clear and simple language, with emphasis on conceptual rather than literal equivalence. Any awkward wording or poor translation choices were identified and discussed to ensure semantic and conceptual accuracy.

The Greek version was then back-translated into English by two professional translators whose native language was English. Translation quality was assessed by comparing the back-translated versions with the original questionnaire in terms of linguistic comparability, interpretive equivalence, and comprehensibility [7]. All translated versions were reviewed and evaluated for semantic and conceptual consistency by an expert committee composed of all translators involved in the process and an independent researcher experienced in questionnaire validation. Any discrepancies were discussed and resolved by consensus.

Pilot testing was conducted with five women who were asked to provide feedback on the clarity of wording and comprehensibility of the questionnaire items. After considering their comments, the expert committee finalized the Greek version of the instrument after making the necessary modifications. Additionally, two supplementary items were added to capture contemporary cervical cancer screening practices related to HPV DNA testing. These questions addressed HPV DNA testing and were analyzed separately from the original domains of the questionnaire. Specifically, the first question was, “Have you ever undergone an HPV DNA test?” and the second question was, “Has your gynecologist ever recommended that you undergo an HPV DNA test?” Responses were dichotomous, with Yes/No answer options.

This addition was based on the growing importance of HPV DNA testing as a primary cervical cancer screening method in contemporary international and European recommendations [10]. The items were designed to capture two complementary aspects: actual uptake of HPV DNA testing and healthcare professional recommendation of the examination. Their inclusion enhanced the contextual relevance of the Greek version without altering the content or scoring of the original items.

Validity

Face and content validity were evaluated as distinct preliminary measurement properties. Face validity was evaluated using qualitative and quantitative methods. In the qualitative phase, five experts were recruited according to predefined criteria, including expertise in cervical cancer, at least five years of professional experience, and familiarity with questionnaire design and validation procedures. Specifically, the expert panel consisted of two gynecologists specializing in cervical cancer, two nurses, and one public health professional, reflecting the importance of cervical cancer as a major public health issue. The mean age of the experts was 52.3 ± 5.5 years, their mean work experience was 17.2 ± 3.5 years, and 80% were male. Individual expert interviews were conducted to assess the questionnaire in terms of item difficulty, ambiguity, potential misinterpretation, clarity of wording, and overall appropriateness of the items, including coherence among items and consistency with the intended purpose of the instrument.

A convenience sample of 15 women from the target population was used for the quantitative assessment of face validity. Participants were asked to rate the questionnaire items for clarity, ambiguity, relevance, comprehensibility, and appropriateness of wording using a 5-point Likert scale ranging from 1 (not important at all) to 5 (extremely important).

Content validity was assessed using the same expert panel to ensure consistency in the evaluation process. Each item was rated by each expert for relevance to the content under study, appropriateness for the target population, and the purpose of the questionnaire. Relevance was rated on a 4-point ordinal scale based on established criteria [11], with response options defined as follows: 1 = not relevant, 2 = somewhat relevant, 3 = quite relevant, and 4 = highly relevant.

Reliability

The reliability of the questionnaire was evaluated using the test-retest method. The questionnaire was administered to 50 women and readministered to the same participants after seven days. This interval was selected to reduce the likelihood that participants would recall their initial responses while remaining short enough to maintain the stability of the construct being measured and minimize the possibility of true changes in cervical cancer prevention knowledge between the two administrations. No educational intervention or feedback was provided between the two administrations. It is important to note that the 15 women who participated in the face validity assessment constituted a separate sample from the 50 women included in the test-retest reliability analysis. No participant took part in both stages of the validation process. The use of independent samples was intended to minimize potential familiarity and recall effects arising from prior exposure to the questionnaire.

**Figure 1:**
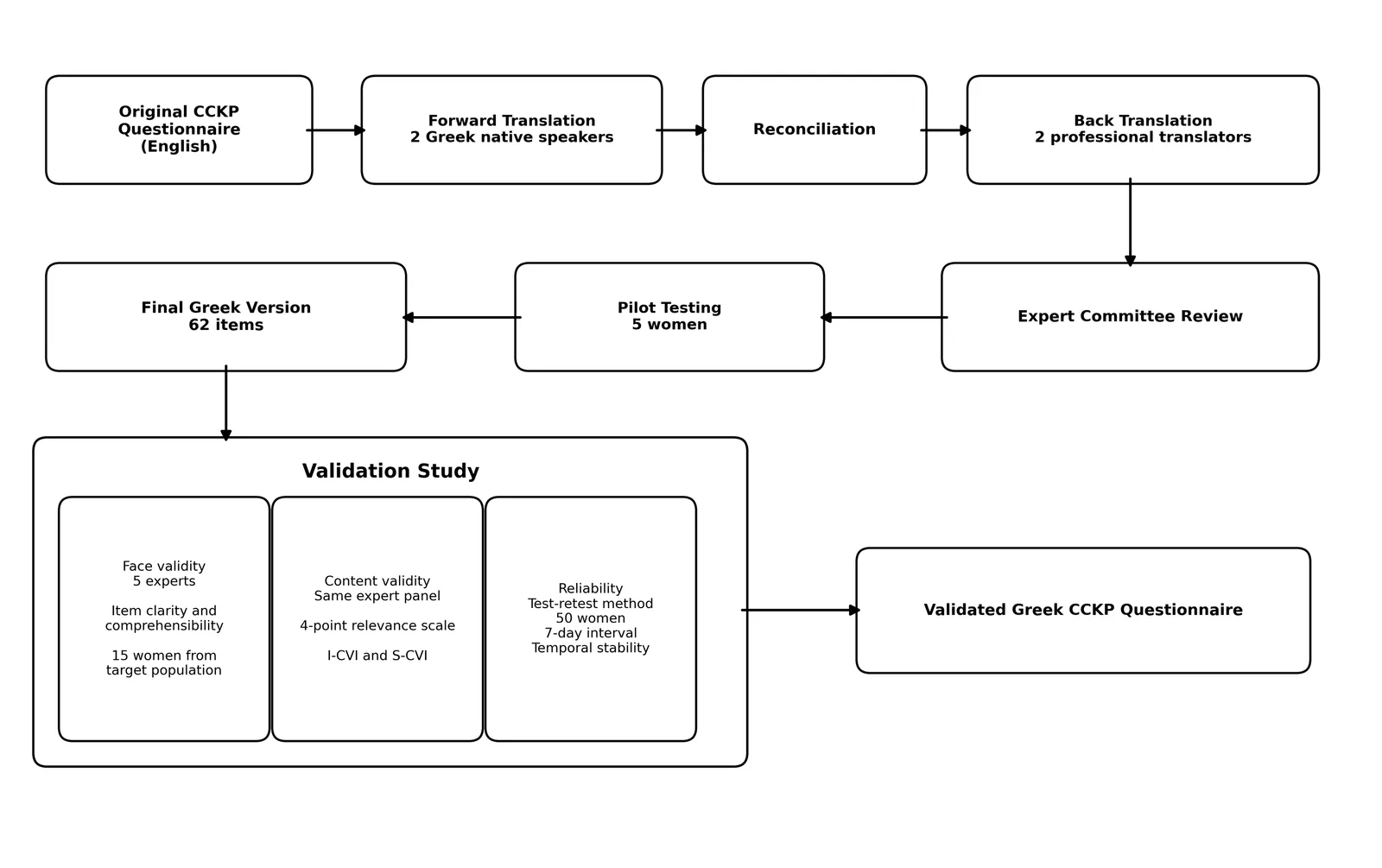
Overview of the study process. CCKP, Cervical Cancer Prevention Knowledge; I-CVI, Item-Level Content Validity Index; S-CVI, Scale-Level Content Validity Index

Figure 1 provides an overview of the study process, outlining the different phases from the initial translation of the questionnaire to its final validation.

Statistical analysis

Quantitative face validity was assessed using the item impact score, calculated as follows:

$\text{Impact score} = \text{Frequency (\%)} \times \mathrm{Importance}$

Items with an impact score of 1.5 or higher were considered acceptable and retained in the questionnaire, whereas items with a score lower than 1.5 were considered for removal [12].

Content validity was assessed using the Content Validity Index (CVI) [13,14]. The Item-Level CVI (I-CVI) was calculated for each item as the proportion of experts who rated the item as either 3 (quite relevant) or 4 (highly relevant) on a 4-point relevance scale [15,16]. An I-CVI value of ≥0.80 was considered acceptable [17]. The Scale-Level CVI (S-CVI) was also calculated as the average of the I-CVI values for all items to determine the overall content validity of the instrument. An S-CVI value greater than 0.90 indicated good content validity [11]. Additionally, universal agreement among the experts was determined based on the number of items with an I-CVI value of 1.00 relative to the total number of questionnaire items. To adjust the I-CVI for chance agreement, the modified kappa statistic was calculated for each item. The probability of chance agreement was estimated according to the number of experts who rated each item as quite relevant or highly relevant. Modified kappa was calculated using the formula:

$k^* = \frac{I\mathrm{-}CVI - P_c}{1 - P_c},$

where Pc represents the probability of chance agreement. Modified kappa values were interpreted as follows: values above 0.74 were considered excellent, values between 0.60 and 0.74 good, and values between 0.40 and 0.59 fair.

Test-retest reliability was assessed using IBM SPSS Statistics for Windows, version 25.0 (released 2017; IBM Corp., Armonk, NY, USA) [18]. Percentage agreement was calculated for all items by comparing the number of identical responses between the two administrations [18]. Test-retest reliability was evaluated using percentage agreement and Cohen’s unweighted kappa coefficient. Unweighted kappa was selected because the response categories used for the reliability analysis were treated as nominal and unordered; therefore, no ordinal distance or weighting between response categories was assumed. Kappa values were interpreted as follows: 0.41-0.60 indicated moderate agreement, 0.61-0.80 substantial agreement, and 0.81-1.00 almost perfect agreement [19]. Internal consistency was assessed separately for each theoretically defined domain. The Kuder-Richardson Formula 20 coefficient was used for dichotomously scored knowledge items, whereas Cronbach’s alpha was calculated for items measured on the 0-5 Likert-type scale.

All questionnaire items were set as mandatory in the electronic survey; therefore, questionnaires could not be submitted unless all required items had been completed. Responses such as “I do not know/I prefer not to answer” were treated as valid response categories rather than missing data. Consequently, no imputation procedures were required.

## Results

Demographic characteristics of the experts and respondents

Table 1 presents the demographic characteristics of the respondents for the quantitative face validity sample and the test-retest reliability sample separately. Among the women who assessed item clarity, relevance, comprehensibility, and wording, the largest proportion were 41-50 years old (46.6%), 73.4% were married, 60% had completed tertiary education, 80% lived in urban areas, 66.7% were employed, and 73.4% had a monthly household income above €1,501. Among the 50 women included in the test-retest reliability assessment, 34% were aged ≤40 years, and an equal proportion were aged ≥51 years. Most participants were married (70%). In terms of educational level, 84% had completed tertiary education. The majority lived in urban areas (80%) and were employed (90%). With respect to monthly household income, the largest proportion reported earnings above €1,501 (44%).

**Table 1:** Demographic characteristics of the respondents. ^*^ Single or separated

Variable	Quantitative face validity sample (n = 15)		Test-retest reliability sample (n = 50)	
	Frequency (n)	Percentage (%)	Frequency (n)	Percentage (%)
Age group				
≤40	4	26.7	17	34
41-50	7	46.6	16	32
≥51	4	26.7	17	34
Marital status				
Married	11	73.4	35	70
Unmarried^*^	2	13.3	14	28
Widow	2	13.3	1	2
Educational level				
Primary education	2	13.3	2	4
Secondary education	4	26.7	6	12
Tertiary education	9	60	42	84
Place of residence				
Urban area	12	80	40	80
Rural area	3	20	10	20
Occupational status				
Employed	10	66.7	45	90
Unemployed	3	20	4	8
Retired	2	13.3	1	2
Monthly family income				
Up to €700	0	0	5	10
€701-€1,000	2	13.3	7	14
€1,001-€1,500	2	13.3	16	32
>€1,501	11	73.4	22	44

Face validity

All five experts considered the tool to be a valuable method for measuring knowledge of cervical cancer. Four of the five experts (n = 4, 80%) found the questionnaire items to be highly relevant, while one expert (n = 1, 20%) disagreed. Two experts (n = 2, 40%) felt that some questions were unnecessary, whereas the remaining three experts (n = 3, 60%) did not support their exclusion. Furthermore, the female respondents who participated in the face validity evaluation confirmed that all items were unambiguous, straightforward, and appropriate with respect to the study objectives. The quantitative face validity results indicated that the impact score for all items exceeded the acceptable threshold of 1.5.

Content validity

According to the results of the content validity assessment, the questionnaire demonstrated satisfactory to excellent relevance at the individual item level (Table 2). Most items had an I-CVI value of 1.00, while the remaining items had an I-CVI value of 0.80, indicating acceptable item-level content validity. For items rated as relevant by all five experts, the I-CVI was 1.00 and the modified kappa was 1.00. For items rated as relevant by four out of five experts, the I-CVI was 0.80, and the modified kappa was 0.76. The S-CVI, calculated as the average of item-level CVI values, was 0.97, indicating excellent overall content validity. The S-CVI/UA was 0.84, indicating universal expert agreement for 84% of the questionnaire items.

**Table 2:** Results for content validity and test-retest reliability. Data are presented as expert relevance ratings, the I-CVI, percentage agreement, and unweighted Cohen’s kappa coefficients. The I-CVI was calculated as the proportion of experts who rated each item as either quite relevant or highly relevant. An I-CVI value of ≥0.80 was considered acceptable. Percentage agreement and unweighted Cohen’s kappa were used to assess test-retest reliability. HPV, human papillomavirus; I-CVI, Item-Level Content Validity Index

Item number	Content validity						Test-retest reliability	
	Expert relevance ratings					I-CVI	Percentage agreement (%)	Unweighted Cohen’s kappa
	Rater 1	Rater 2	Rater 3	Rater 4	Rater 5			
General knowledge about cervical cancer								
1	4	4	4	4	4	1.00	70.0	0.68
2	4	4	4	4	4	1.00	78.0	0.67
3	2	3	3	3	4	0.80	68.0	0.57
4	4	4	4	4	4	1.00	72.0	0.63
5	3	4	3	4	4	1.00	68.0	0.59
6	4	4	4	4	4	1.00	90.0	0.84
Risk factors								
1	4	4	4	4	4	1.00	90.0	0.84
2	4	4	4	4	4	1.00	62.0	0.58
3	4	4	4	4	4	1.00	68.0	0.56
4	4	4	4	4	4	1.00	72.0	0.63
5	4	4	4	4	4	1.00	98.0	0.84
6	4	4	4	4	4	1.00	88.0	0.78
7	4	4	4	4	4	1.00	98.0	0.88
8	4	4	4	4	4	1.00	84.0	0.75
9	3	2	4	4	4	0.80	76.0	0.68
10	4	4	4	4	4	1.00	92.0	0.84
11	4	4	4	4	4	1.00	98.0	0.92
12	4	4	4	4	4	1.00	84.0	0.75
13	4	4	4	4	4	1.00	76.0	0.72
14	3	3	4	4	4	1.00	84.0	0.78
15	4	4	4	4	4	1.00	74.0	0.62
16	3	4	3	4	4	1.00	88.0	0.79
17	3	4	3	4	4	1.00	74.0	0.69
Knowledge about primary prevention								
A. Lifestyle								
1	4	4	4	4	4	1.00	74.0	0.63
2	4	4	4	4	4	1.00	76.0	0.66
3	4	4	4	4	4	1.00	84.0	0.72
4	4	4	4	4	4	1.00	68.0	0.62
5	4	4	4	4	4	1.00	74.0	0.68
6	3	2	4	4	4	0.80	86.0	0.77
7	4	2	3	4	4	0.80	80.0	0.78
8	4	2	3	4	4	0.80	84.0	0.74
B. Vaccine								
9	4	4	4	4	4	1.00	80.0	0.72
10	4	4	4	4	4	1.00	90.0	0.75
11	4	4	4	4	4	1.00	96.0	0.85
12	4	4	4	4	4	1.00	96.0	0.83
13	4	4	4	4	4	1.00	98.0	0.89
14	3	3	4	2	4	0.80	98.0	0.94
C. Optimal age for HPV vaccination								
15	4	4	4	4	4	1.00	92.0	0.81
Secondary prevention								
A. Distressing symptoms								
1	4	4	4	4	4	1.00	88.0	0.80
2	4	4	4	4	4	1.00	78.0	0.72
3	4	4	4	4	4	1.00	96.0	0.84
4	4	4	4	4	4	1.00	80.0	0.70
5	4	4	4	4	4	1.00	92.0	0.88
6	4	4	4	4	4	1.00	84.0	0.82
7	4	4	4	4	4	1.00	76.0	0.72
8	4	4	4	4	4	1.00	78.0	0.73
9	4	4	4	4	4	1.00	68.0	0.62
B. Cytological examination								
10	4	4	4	4	4	1.00	82.0	0.75
11	3	2	4	4	4	0.80	74.0	0.69
12	4	4	4	4	4	1.00	88.0	0.78
13	4	4	4	4	4	1.00	70.0	0.67
14	4	4	4	4	4	1.00	70.0	0.67
15	4	4	4	4	4	1.00	78.0	0.72
16	4	4	4	4	4	1.00	74.0	0.70
17	2	3	4	4	4	0.80	86.0	0.80
18	2	3	4	4	4	0.80	84.0	0.77
19	4	4	4	4	4	1.00	68.0	0.62
20	4	4	4	4	4	1.00	98.0	0.97
21	4	4	4	4	4	1.00	72.0	0.69
Sources of information about cervical cancer								
1	4	4	4	4	4	1.00	76.0	0.72
HPV DNA test								
1	4	4	4	4	4	1.00	74.0	0.69
2	3	2	4	4	4	0.80	78.0	0.72

Reliability

Results from the test-retest reliability analysis showed that percentage agreement ranged from 62.0% to 98.0%, while unweighted Cohen’s kappa values ranged from 0.56 to 0.97. According to commonly used interpretation criteria, the kappa values indicated moderate to almost perfect agreement. Items with lower kappa values within the moderate agreement range were observed only among selected items in the general knowledge and risk-factor domains. These items were retained because their percentage agreement was acceptable, their kappa values did not indicate poor agreement, and their content validity indices met the predefined acceptability criterion. Nevertheless, these items should be further evaluated in future studies with larger samples. Overall, the findings suggest acceptable temporal stability, with item-level agreement ranging from moderate to almost perfect.

All 15 face validity questionnaires and all 50 test-retest questionnaires were complete and were included in the respective analyses. No questionnaires were excluded due to missing data, and no imputation was performed.

Table 3 presents the internal consistency results of the questionnaire. All coefficients indicate acceptable levels of internal consistency.

**Table 3:** Internal consistency of the questionnaire. ^*^ KR-20 ^#^ Cronbach’s alpha HPV, human papillomavirus

Domain	Internal consistency
General knowledge about cervical cancer	0.742^*^
Risk factors	0.884^#^
Knowledge about primary prevention	0.730^*^
Secondary prevention	0.862^*^
Sources of information about cervical cancer	N/A
HPV DNA test	N/A

## Discussion

The objective of the present study was to translate, culturally adapt, and preliminarily evaluate the Greek version of the CCKP questionnaire. The findings provide preliminary evidence that the adapted questionnaire is understandable and relevant to the target population and demonstrates item-level temporal stability ranging from moderate to almost perfect.

The translation and adaptation process used in the current study was based on established recommendations [6,7]. In this regard, both conceptual and linguistic equivalence of the instrument were achieved through the systematic participation of the expert group and the back-translation process, which was considered necessary to ensure the cultural sensitivity of the questions included in the questionnaire.

Findings from the face and content validity assessments confirmed that the questionnaire was clear, comprehensible, and valid for measuring the intended content. The I-CVI, S-CVI/Ave, S-CVI/UA, and modified kappa findings provide evidence of content validity and expert agreement regarding item relevance. These findings are consistent with previous methodological studies emphasizing the importance of content validity in instrument development and adaptation [15,16,20].

The reliability of the instrument was assessed using a test-retest approach, demonstrating adequate temporal stability with kappa values ranging from moderate to almost perfect according to Cohen’s criteria. This indicates sufficient consistency in measurement and supports its potential use in research and practice. These findings are consistent with methodological recommendations emphasizing the combined assessment of agreement beyond chance and internal consistency when evaluating measurement instruments [18,21]. Although most items demonstrated substantial to almost perfect agreement, four items showed moderate test-retest reliability, with kappa values ranging from 0.56 to 0.59. These items were retained in the Greek version because they met the predefined content validity criteria and did not demonstrate poor agreement. The lower kappa values may reflect variability in respondents’ knowledge or uncertainty regarding specific aspects of cervical cancer prevention rather than instability of the questionnaire itself. Nevertheless, these items should be interpreted with caution and further examined in larger validation studies.

Within the Greek population, research examining knowledge and attitudes toward cervical cancer is scarce and is usually based on local samples only [22,23]. At the same time, evidence indicates considerable gaps in knowledge and inadequate attitudes regarding cancer screening and vaccination among Greek women [24]. Unfortunately, the literature lacks information on the psychometric validation of such instruments in this field, highlighting the contribution of the present preliminary validation study.

The modification of the questionnaire to suit the linguistic, cultural, and healthcare context of the Greek population through the inclusion of two supplementary HPV DNA testing items improved the comprehensiveness and contextual appropriateness of the tool without compromising its fundamental framework. This approach has been recommended by previous studies, which suggest that adapting measurement tools for different populations is critical to ensuring the maintenance of conceptual equivalence [6,7,20,25].

Limitations

Despite the promising findings, this study has several limitations. First, the use of nonprobability snowball sampling may have introduced selection bias and limited the representativeness and generalizability of the findings. Second, only a small number of women participated in the pilot testing phase, which may have restricted the breadth of feedback regarding item clarity and comprehensibility. Third, the expert panel consisted of only five members, which may have limited the stability of the content validity estimates, although modified kappa was calculated to account for chance agreement. In addition, the two supplementary HPV DNA testing items assessed screening behavior and healthcare-professional recommendation rather than knowledge and should therefore be interpreted separately from the original questionnaire domains.

Although unweighted Cohen’s kappa was used to assess agreement beyond chance, 95% confidence intervals for the kappa estimates were not calculated. Therefore, the precision of the test-retest reliability findings should be interpreted with caution. Further research using larger and more diverse samples is required to comprehensively evaluate the psychometric properties of the Greek version of the CCKP questionnaire, including its construct validity, criterion validity, and factor structure.

## Conclusions

The Greek version of the CCKP questionnaire demonstrated satisfactory preliminary measurement properties, including acceptable face validity, content validity, test-retest reliability, and internal consistency. The structured translation and cross-cultural adaptation process resulted in a comprehensible and contextually relevant instrument for assessing cervical cancer prevention knowledge among women in Greece. However, the two supplementary HPV DNA testing items should be interpreted separately from the original questionnaire domains, and their effect on scoring and comparability with the original instrument requires further investigation.

This instrument may help address the limited availability of validated Greek-language measures for assessing cervical cancer prevention knowledge and may support future research and educational interventions. Its use may also contribute to the identification of knowledge gaps and inform the development of targeted prevention and health information initiatives for women. Further studies involving larger and more diverse samples are needed to evaluate the broader psychometric properties of the questionnaire, including its construct validity, criterion validity, and factor structure.
